# Supplementary material for: Whole-slide mapping of tumor tissue fiber architecture via computational scattered light imaging
Source: Biomed Opt Express. 2026 Apr 29;17(5):2675–91. doi: 10.1364/BOE.595972 (PMC13178623; doi:10.1364/BOE.595972)
Supplement: Supplementary file 1 [file boe-17-5-2675-s001.pdf]

Supplemental Document

Biomedical Optics EXPRESS

Whole-slide mapping of tumor tissue fiber architecture via computational scattered light imaging: supplement

HAMED ABBASI,[1,2,*] LOES ETTEMA,[1] RENS VAN ELK,[1] MEIKE ESKES,[1] MICHAIL DOUKAS,[3] SJORS A. KOPPES,[3] STIJN KEEREWEER,[2] AND MIRIAM MENZEL[1,4]

[1]*Department of Imaging Physics, Faculty of Applied Sciences, Delft University of Technology, Delft, The Netherlands*
[2]*Department of Otorhinolaryngology and Head and Neck Surgery, Erasmus MC Cancer Institute, University Medical Center Rotterdam, Rotterdam, The Netherlands*
[3]*Department of Pathology, Erasmus MC, University Medical Center Rotterdam, Rotterdam, The Netherlands*
[4]*m.menzel@tudelft.nl*
[*]*h.abbasi@tudelft.nl*

This supplement published with Optica Publishing Group on 29 April 2026 by The Authors under the terms of the Creative Commons Attribution 4.0 License in the format provided by the authors and unedited. Further distribution of this work must maintain attribution to the author(s) and the published article's title, journal citation, and DOI.

Supplement DOI: https://doi.org/10.6084/m9.figshare.32034363

Parent Article DOI: https://doi.org/10.1364/BOE.595972

Supplemental Document

Whole-Slide Mapping of Tumor Tissue Fiber Architecture via Computational Scattered Light Imaging

HAMED ABBASI,[1,2,*] LOES ETTEMA,[1] RENS VAN ELK,[1] MEIKE ESKES,[1] MICHAIL DOUKAS,[3] SJORS A. KOPPES,[3] STIJN KEEREWEER,[2] MIRIAM MENZEL[1,4]

[1]*Department of Imaging Physics, Faculty of Applied Sciences, Delft University of Technology, Delft, the Netherlands.*
[2]*Department of Otorhinolaryngology and Head and Neck Surgery, Erasmus MC Cancer Institute, University Medical Center Rotterdam, Rotterdam, the Netherlands.*
[3]*Department of Pathology, Erasmus MC, University Medical Center Rotterdam, Rotterdam, the Netherlands.*
**h.abbasi@tudelft.nl*
[4]*m.menzel@tudelft.nl*

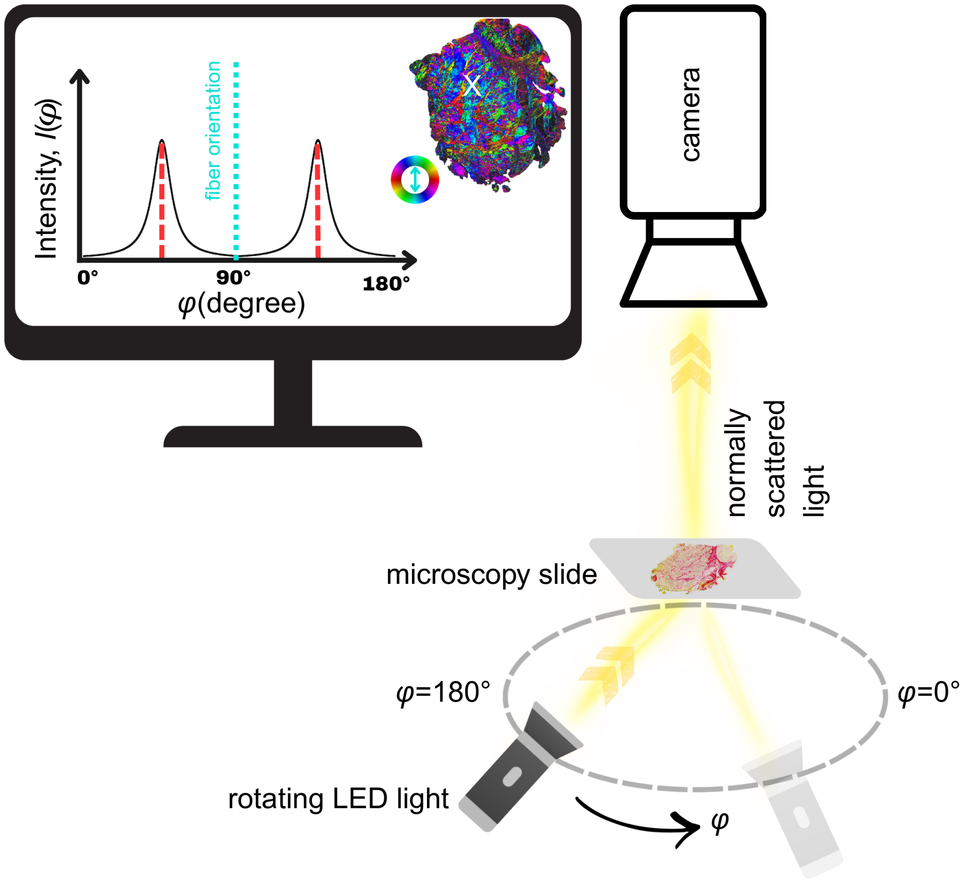

Fig. S1. ComSLI setup. A rotating LED lamp illuminates the tissue section from below under a fixed polar angle of about 45°. A camera records the normally scattered light for different rotation angles. The intensity of each image pixel in the resulting image series, $I(\varphi)$, is evaluated. The monitor shows an exemplary intensity profile of one image pixel; the in-plane fiber orientation is determined by the mid-position of a peak pair and displayed according to a color wheel (red: $\varphi = 0°$, yellow: 30°, green: 60°, cyan: 90°, blue: 120°, magenta: 150°). In the given example the fiber orientation is φ=90° and shown in cyan.

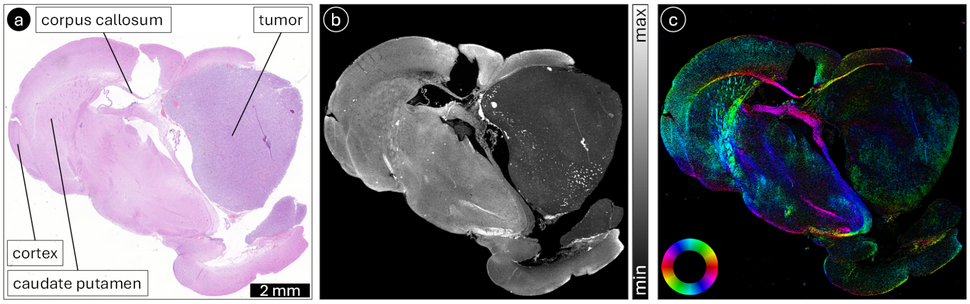

Fig. S2. ComSLI in an additional mouse glioma brain section (H&E-stained, 4-µm-thin, FFPE). (a) Bright-field image with tumor and selected anatomical regions labeled. (b) Average scattering map. (c) Fiber orientation map, color-coded according to the color wheel in the bottom left (red: $\varphi = 0°$, yellow: 30°, green: 60°, cyan: 90°, blue: 120°, magenta: 150°).

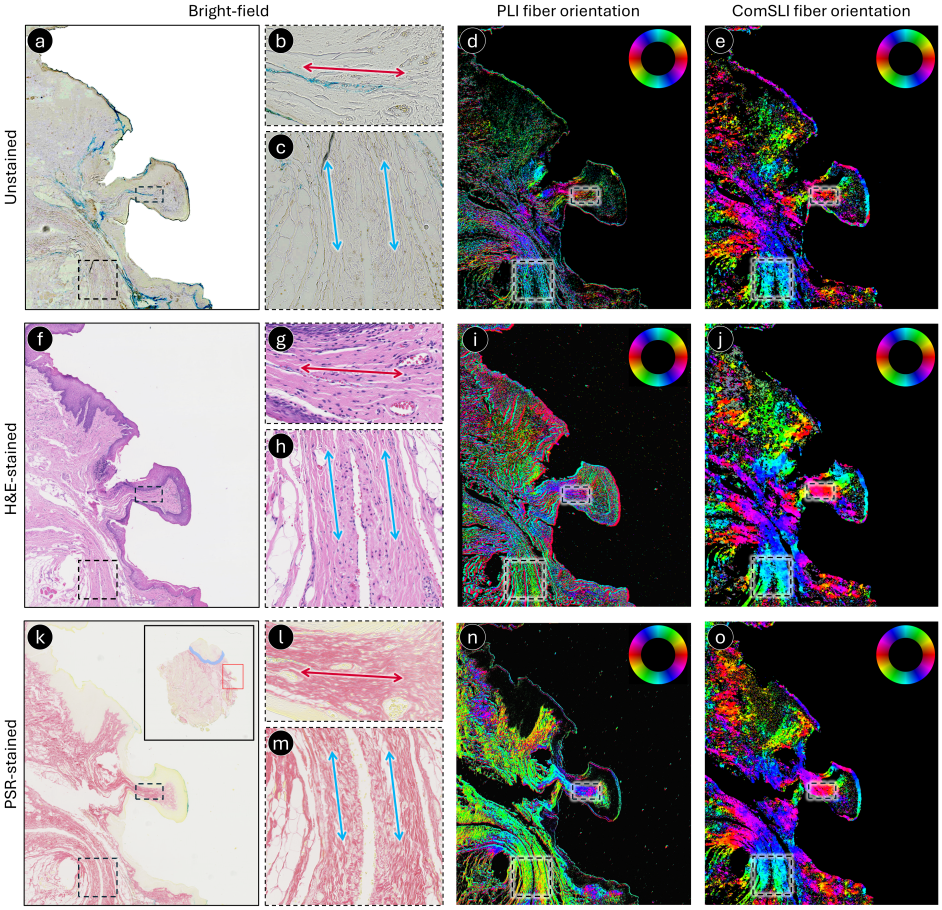

Fig. S3. Polarized light imaging (PLI) versus ComSLI in FFPE 4-µm-thin collagen-rich human tongue sections: unstained (a–e), H&E-stained (f–j), and PSR-stained (k–o). Panels (a), (f), and (k) show bright-field digital microscopy images of the unstained, H&E-stained, and PSR-stained sections, respectively. In (k), the whole-slide PSR-stained bright-field digital microscopy image is shown as an inset, with the zoom-in area indicated by a red rectangle and the tumor boundary highlighted in blue (same section as in Fig. 5b). Panels (b), (g), and (l) show areas dominated by horizontal collagen fibers (as indicated by red arrows), which are expected to appear red in the fiber orientation maps. Panels (c), (h), and (m) show areas dominated by vertical fibers (as indicated by cyan arrows), which are expected to appear cyan in the fiber orientation maps. Panels (d), (i), and (n) present fiber orientation maps obtained with PLI (corresponding to the slow-axis orientations), and panels (e), (j), and (o) show fiber orientation maps obtained using ComSLI. All fiber orientation maps are color-coded according to the color wheel shown in the top right (red: φ = 0°, yellow: 30°, green: 60°, cyan: 90°, blue: 120°, magenta: 150°). In the fiber orientation maps, pixels with low retardance or average scattering (as in the empty-slide area) are shown in black. While ComSLI correctly shows fiber orientation for both horizontal and vertical fibers, PLI shows the correct orientation only in the unstained section.

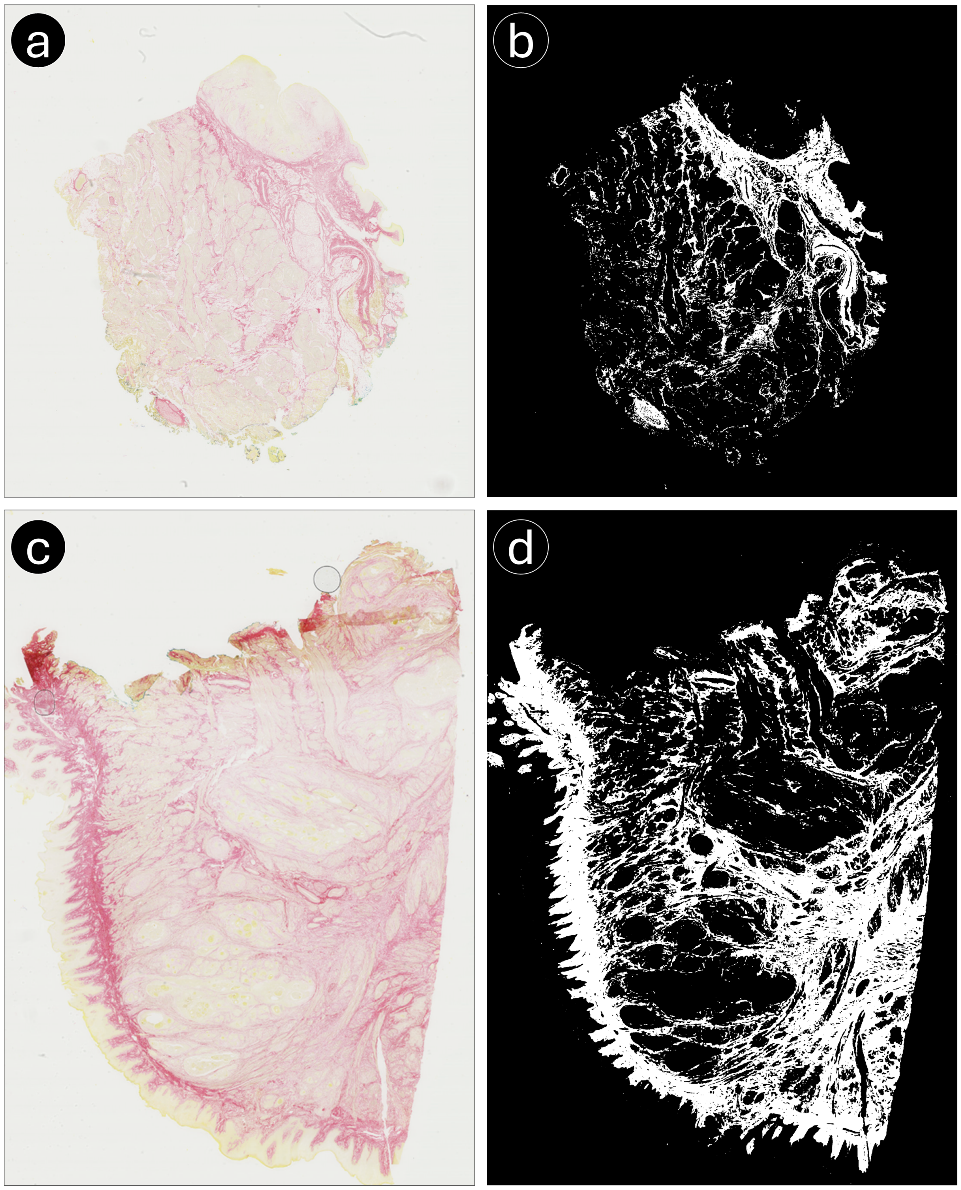

Fig S4. High-resolution whole-slide images of the PSR-stained tongue sections from main Fig. 5: Bright-field digital microscopy images (left) and corresponding collagen fiber masks (right) for the low-WPOI sample (top) and high-WPOI sample (bottom).

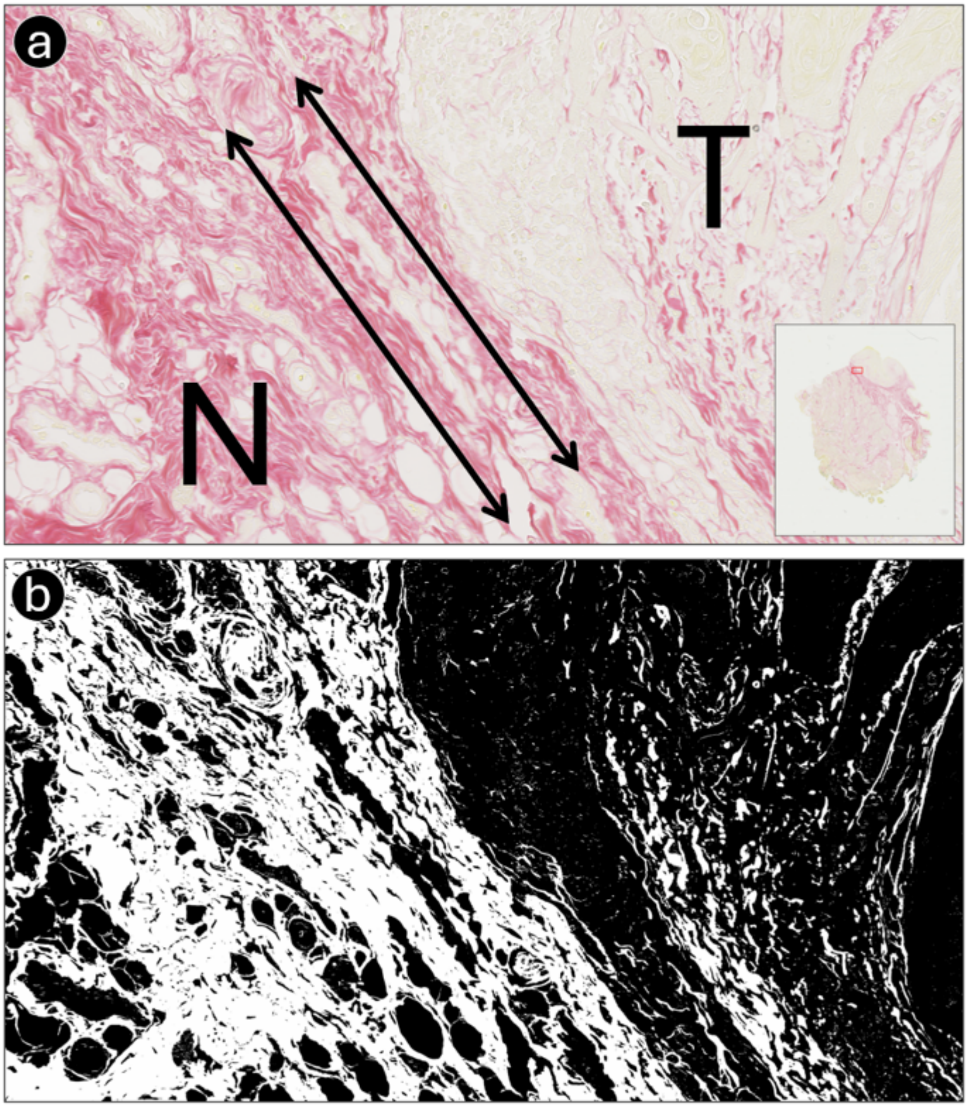

Fig S5. Zoom-in of the low-WPOI section at the left tumor boundary. (a) High-resolution bright-field digital microscopy image of PSR-stained section (see inset). (b) Corresponding high-resolution collagen fiber mask. The tumor area is annotated by “T” and the normal area by “N”. Arrows indicate the dominant fiber orientations (parallel to the tumor boundary).

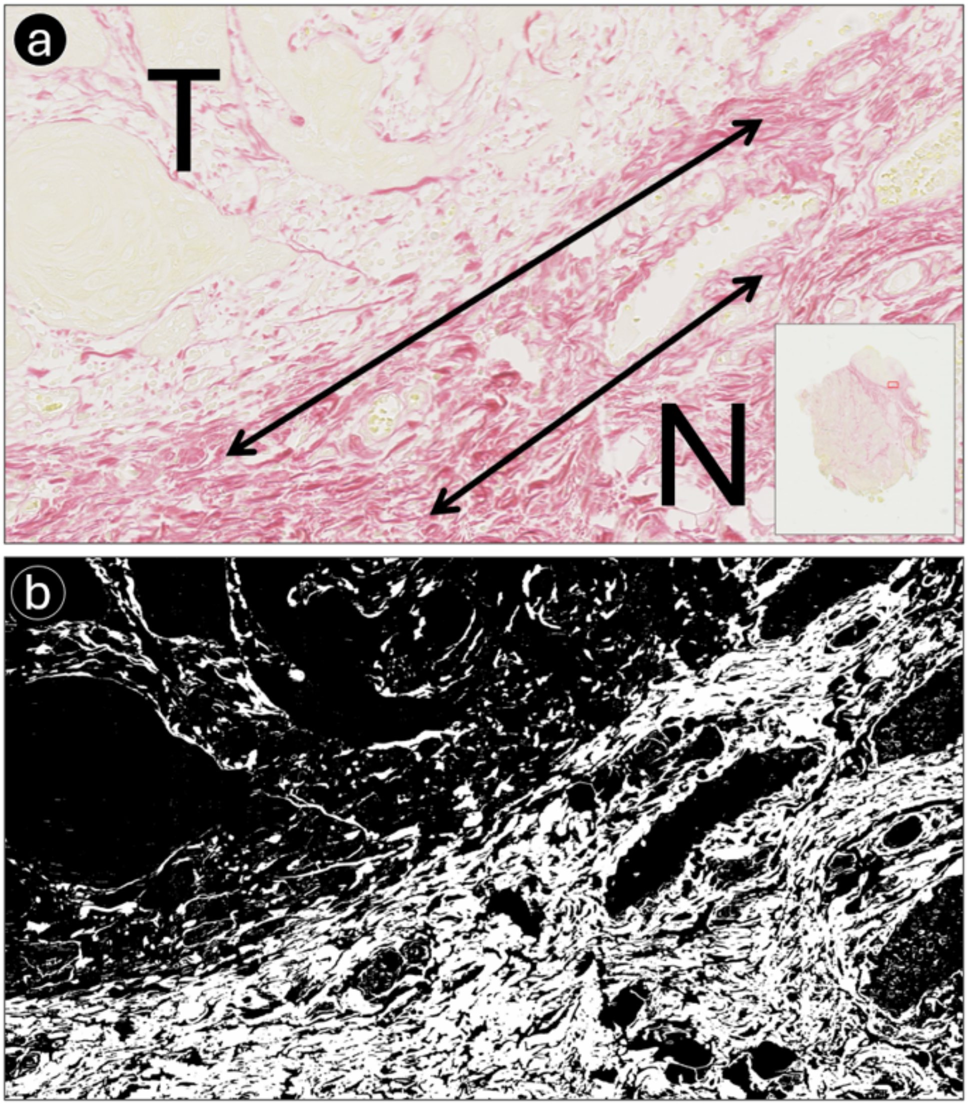

Fig S6. Zoom-in of the low-WPOI section at the right tumor boundary. (a) High-resolution bright-field digital microscopy image of PSR-stained section (see inset). (b) Corresponding high-resolution collagen fiber mask. The tumor area is annotated by “T” and the normal area by “N”. Arrows indicate the dominant fiber orientations (parallel to the tumor boundary).

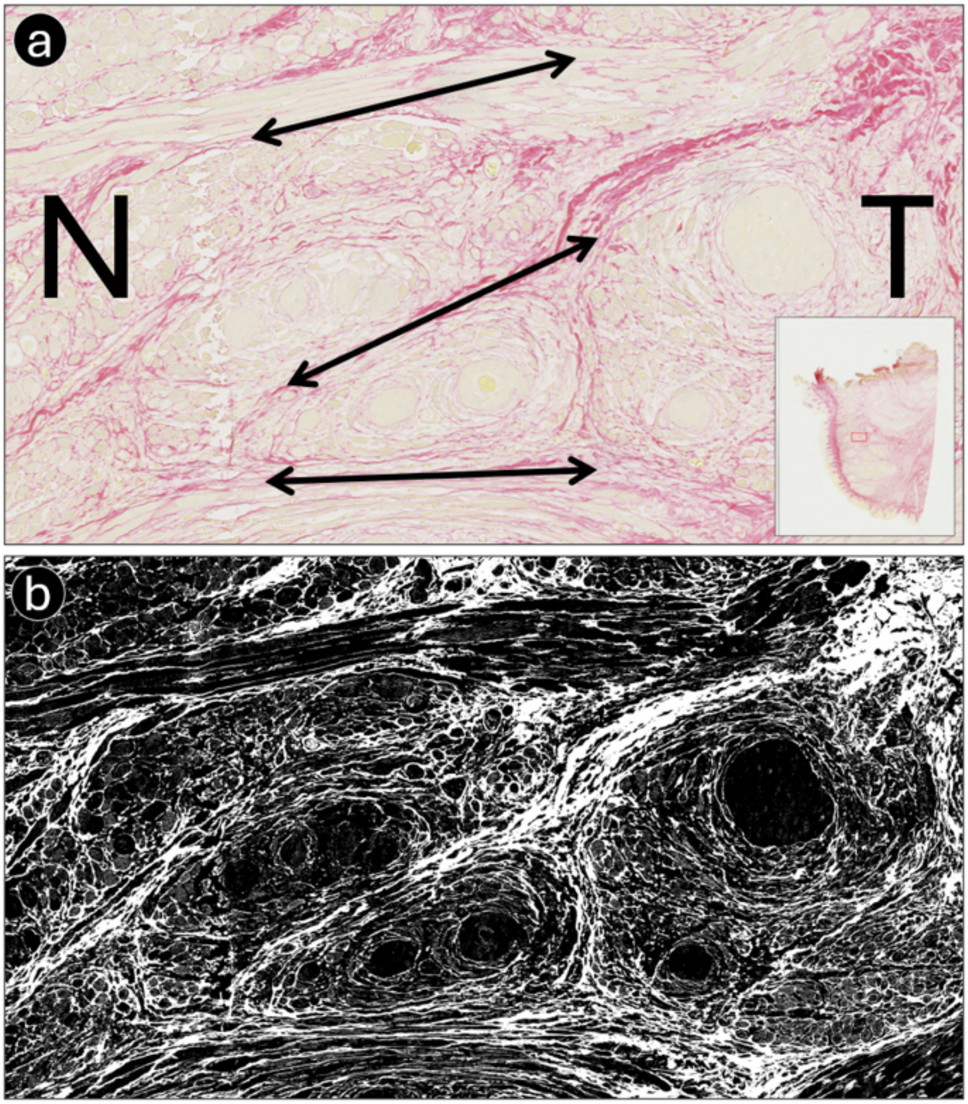

Fig S7. Zoom-in of the high-WPOI section at the tumor boundary (bottom part). (a) High-resolution bright-field digital microscopy image of PSR-stained section (see inset). (b) Corresponding high-resolution collagen fiber mask. The tumor area is annotated by “T” and the normal area by “N”. Arrows indicate the dominant fiber orientations (perpendicular to the tumor boundary).

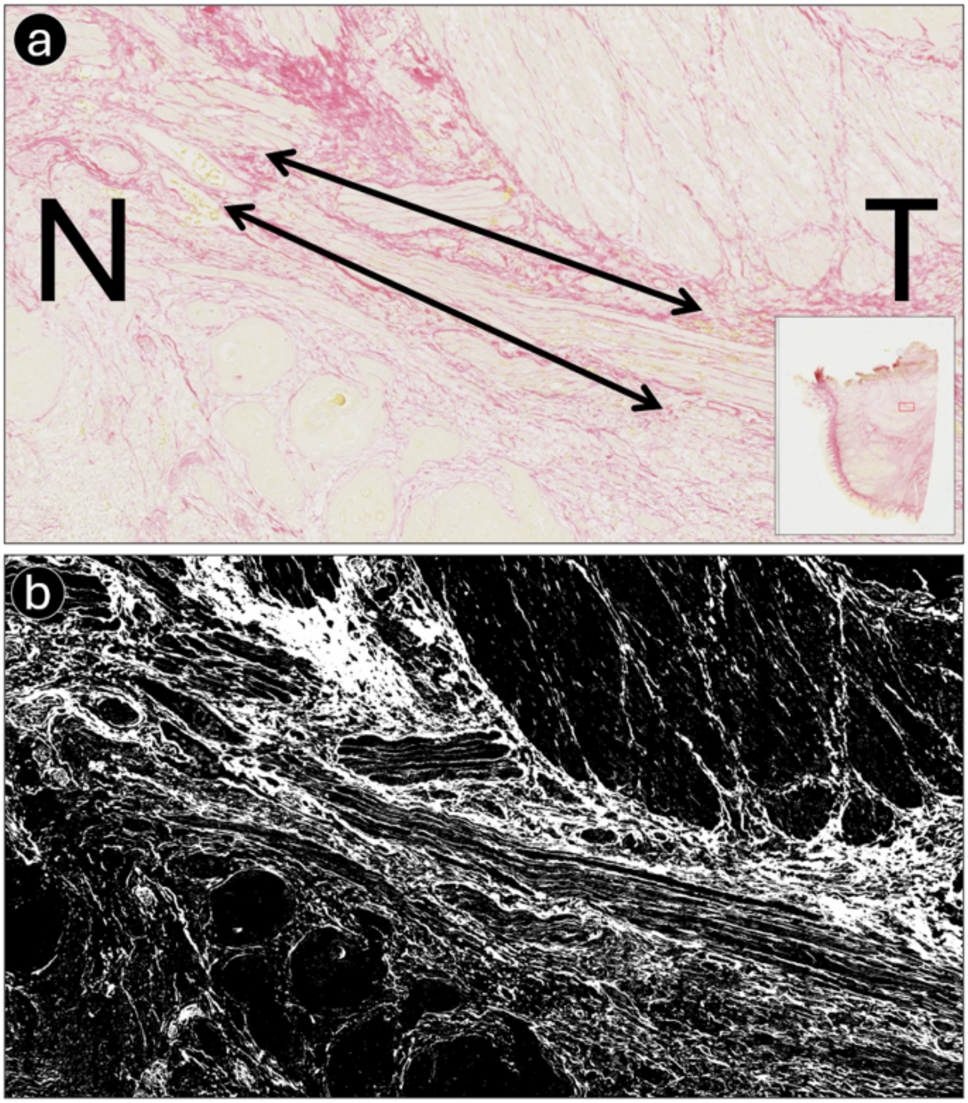

Fig S8. Zoom-in of the high-WPOI section at the tumor boundary (top part). (a) High-resolution bright-field digital microscopy image of PSR-stained section (see insert). (b) Corresponding high-resolution collagen fiber mask. The tumor area is annotated by "T" and the normal area by "N". Arrows indicate the dominant fiber orientations (perpendicular to the tumor boundary).
